# Radiomics in differential diagnosis of Wilms tumor and neuroblastoma with adrenal location in children

**DOI:** 10.1007/s00330-024-10589-8

**Published:** 2024-02-05

**Authors:** Ilker Ozgur Koska, H. Nursun Ozcan, Aziz Anil Tan, Beyza Beydogan, Gozde Ozer, Berna Oguz, Mithat Haliloglu

**Affiliations:** 1 Department of Radiology, Behcet Uz Children’s Hospital, Konak İzmir, Turkey; 2 Department of Radiology, Hacettepe University School of Medicine, Ankara, Turkey; 3 Department of Radiology, Sincan Training and Research Hospital, Ankara, Turkey

**Keywords:** Abdomen, Neuroblastoma, Wilms tumor, Computed tomography, Machine learning

## Abstract

**Objectives:**

Machine learning methods can be applied successfully to various medical imaging tasks. Our aim with this study was to build a robust classifier using radiomics and clinical data for preoperative diagnosis of Wilms tumor (WT) or neuroblastoma (NB) in pediatric abdominal CT.

**Material and methods:**

This is a single-center retrospective study approved by the Institutional Ethical Board. CT scans of consecutive patients diagnosed with WT or NB admitted to our hospital from January 2005 to December 2021 were evaluated. Three distinct datasets based on clinical centers and CT machines were curated. Robust, non-redundant, high variance, and relevant radiomics features were selected using data science methods. Clinically relevant variables were integrated into the final model. Dice score for similarity of tumor ROI, Cohen’s kappa for interobserver agreement among observers, and AUC for model selection were used.

**Results:**

A total of 147 patients, including 90 WT (mean age 34.78 SD: 22.06 months; 43 male) and 57 NB (mean age 23.77 SD:22.56 months; 31 male), were analyzed. After binarization at 24 months cut-off, there was no statistically significant difference between the two groups for age (*p* = .07) and gender (*p* = .54). CT clinic radiomics combined model achieved an F1 score of 0.94, 0.93 accuracy, and an AUC 0.96.

**Conclusion:**

In conclusion, the CT-based clinic-radiologic-radiomics combined model could noninvasively predict WT or NB preoperatively. Notably, that model correctly predicted two patients, which none of the radiologists could correctly predict. This model may serve as a noninvasive preoperative predictor of NB/WT differentiation in CT, which should be further validated in large prospective models.

**Clinical relevance statement:**

CT-based clinic-radiologic-radiomics combined model could noninvasively predict Wilms tumor or neuroblastoma preoperatively.

**Key Points:**

• *CT radiomics features can predict Wilms tumor or neuroblastoma from abdominal CT preoperatively.*

• *Integrating clinic variables may further improve the performance of the model.*

• *The performance of the combined model is equal to or greater than human readers, depending on the lesion size.*

**Supplementary Information:**

The online version contains supplementary material available at 10.1007/s00330-024-10589-8.

## Introduction

The most common extracranial solid tumor in childhood is neuroblastoma (NB). Approximately one-third of NB originates from the adrenal gland [1]. Wilms tumor (WT) is the most common renal tumor in childhood and typically presents as an abdominal mass [2, 3]. These tumors exhibit vastly different clinical course and management strategies. Thus, early distinction is mandatory.

MRI is the preferred modality for the assessment of NB and WT. However, anesthesia is required for MRI, and some surgeons, including the ones in our institute, may prefer preoperative CT prior to surgery. When the tumor size is considerable, which is the usual case with childhood intraabdominal solid tumors, identifying the organ of origin and actual tumor type becomes increasingly tricky [3–6].

Contrary to North America, European guidelines prefer preoperative chemotherapy and avoid biopsy for WT management since surgery becomes easier, tumor spillage decreases, and downgrading of the tumor, which eventually leads to less radiotherapy requirement, can be a possibility [3, 5]. On the other hand, NB responds well to neoadjuvant chemotherapy, which is usually followed by surgery for local disease control in conjunction with radiotherapy. The chemotherapy protocols are quite different for both tumors, and a misleading diagnosis may lead to inappropriate chemotherapy regimens administration. Therefore, preoperative differentiation between WT and NB is essential for applying appropriate chemotherapy regimens and optimal management of these patients [5, 6].

However, without pathologic specimens using only classical imaging findings, differentiation of these tumors yields an 86% global accuracy [7, 8]. In suspected NB cases, collecting urinary catecholamines may additionally help [9]. Nevertheless, more accurate, reliable, reproducible, and automatized methods are needed to differentiate NB and WT.

Machine learning methods, including radiomics and deep learning, have recently gained considerable attention to quantitively assess medical imaging data. Successful applications of radiomics for WT stage determination, NB MYCN proto-oncogene (MYCN) status determination, and pathologic type determination are published in the literature [10–14].

To our knowledge, no machine learning study has been applied to differentiate WT and NB from preoperative CT images. Our aim in this study was to differentiate NB and WT from the preoperative CT scans using machine learning.

## Methods

### Patients

**Fig. 1:**
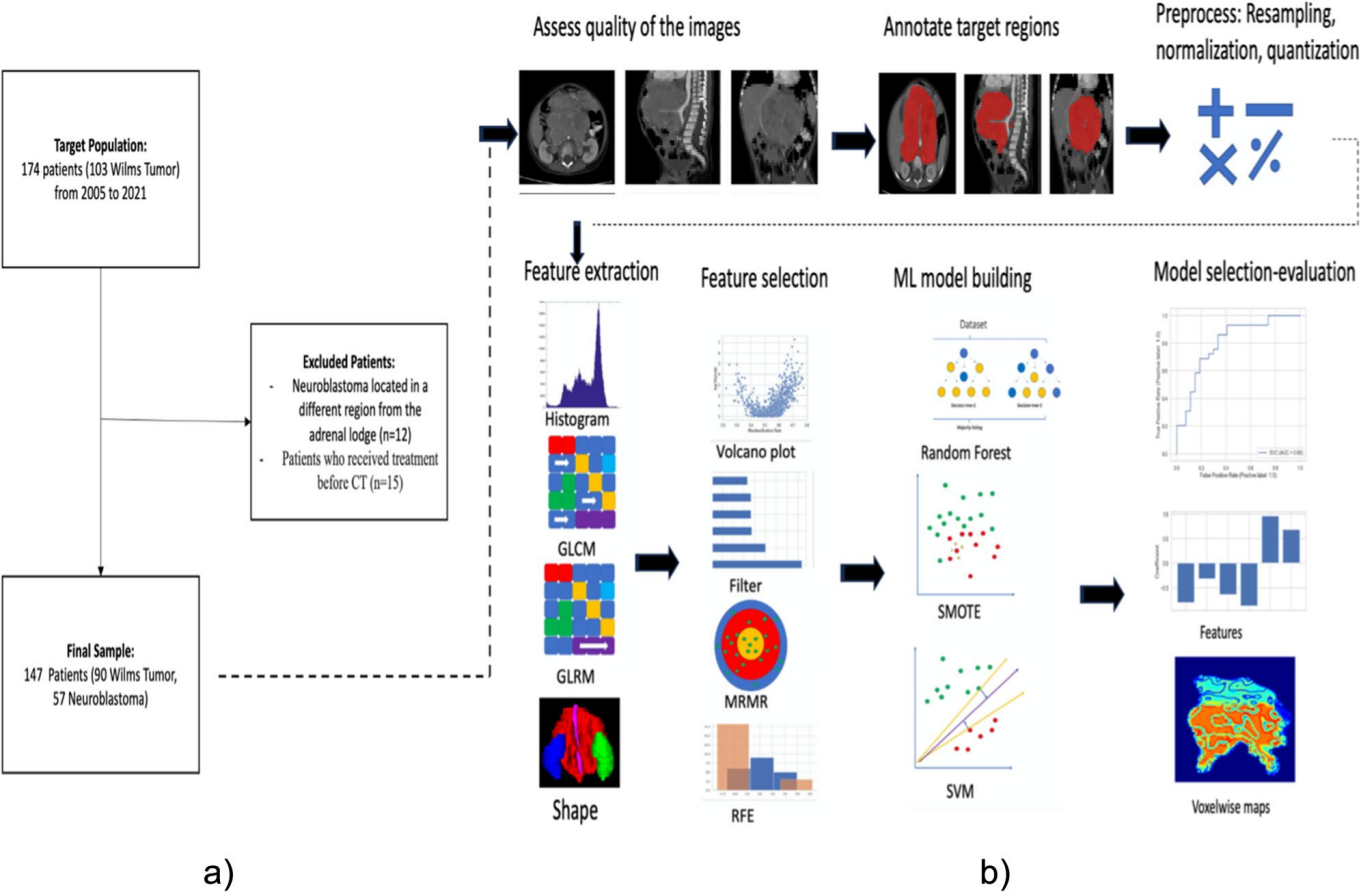
Overview of patient selection and study pipeline. **a** Patient selection flow. **b** Study pipeline

This is a single-center retrospective study approved by the Institutional Ethical Board (GO 21/1282). The informed patient consent was waived by the institutional Ethics Committee due to the retrospective nature of the study. CT scans of consecutive patients diagnosed with NB and WT admitted to our hospital from January 2005 to December 2021 were evaluated. Patients who received treatment before CT were excluded from the study. A total of 147 patients with pathologically confirmed diagnoses were included. The inclusion and exclusion criteria are presented in Fig. 1.

### CT protocol

In our institution, CT images were obtained after administering 2 cc/kg of iodinated non-ionic intravenous contrast material by an automatic injector at a 2–3 mL/s rate. All CTs -including external centers- were performed during the portal venous phase. The distribution of the slice thickness of the CT scans was as follows: 1.25 mm, 2 mm, 2.5 mm, 3 mm, and 5 mm. The kV settings and slice thickness distributions were provided in the supplementary material (Figure S1, Figure S2).

### Clinic-radiologic data collection

Age information is dichotomized using 24 months as the cut-off. Vomiting, diarrhea, constipation, abdominal distension, fever, abdominal mass, pain, and incidental detection were noted as clinical variables. Three distinct datasets were curated. For the first dataset, the patients were separated according to the institute’s origin. The images of the patients acquired in our institute comprised the development set, and the patients whose images were acquired in different institutes, referred to our hospital, and loaded to our local PACS comprised the external validation set; for the second and third datasets, CT machine-based partition was applied. For the second dataset, the images acquired by any machine, excluding Siemens, were used as a development set, and the Siemens images were used as an external validation set. In the third dataset, images acquired in any machine, excluding GE, were used as a development set, and the images acquired with GE CT machines were used as an external validation set. CT machines used in this study are listed in the supplementary material.

### Ground truth segmentation, human reader study, and image labelling

**Fig. 2:**
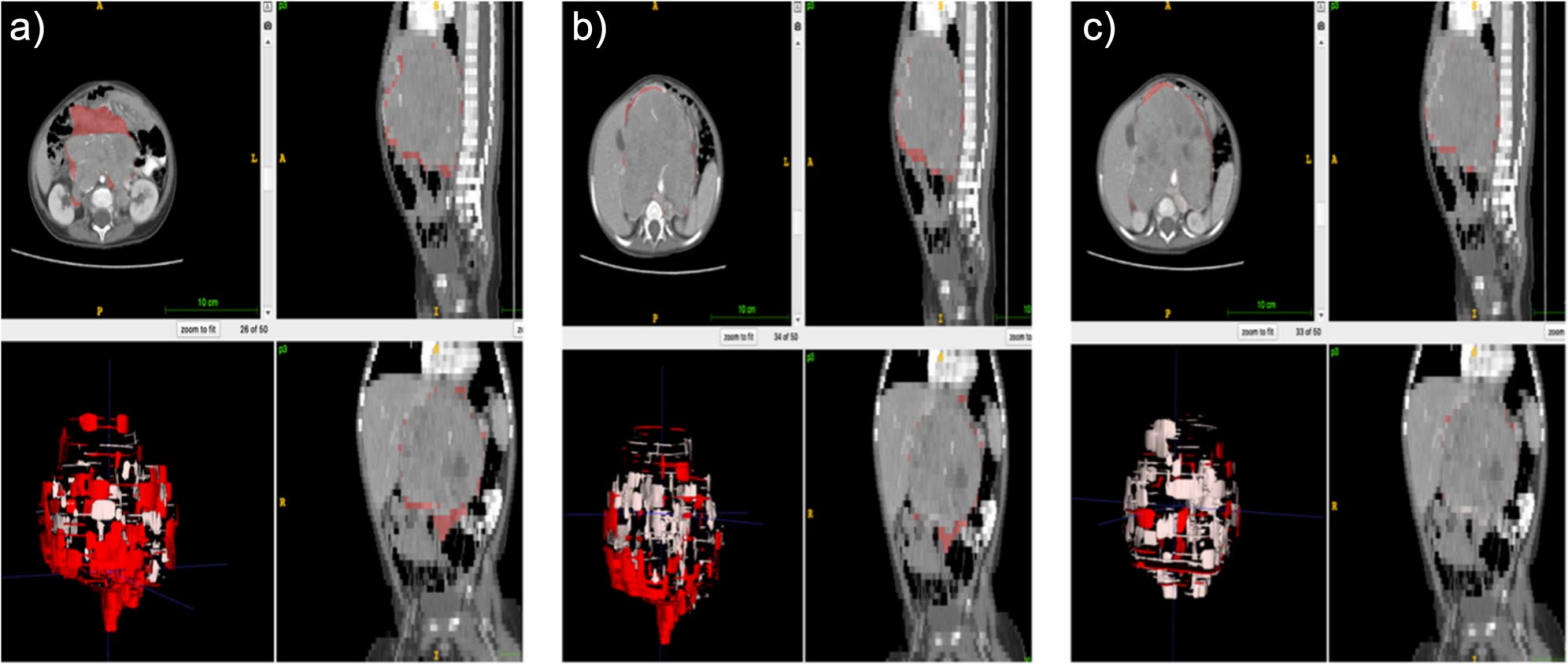
Difference maps overlaid on images. The lower left corner is a 3D mesh of the error map. Red represents oversegmented; pink represents undersegmented regions compared to the reference. The other three corners are axial, sagittal, and coronal single-slice overlays. **a** Error map of observer 1 relative to observer 2. **b** Error map of observer 1 relative to observer 3. **c** Error map of observer 2 relative to observer 3

**Fig. 3:**
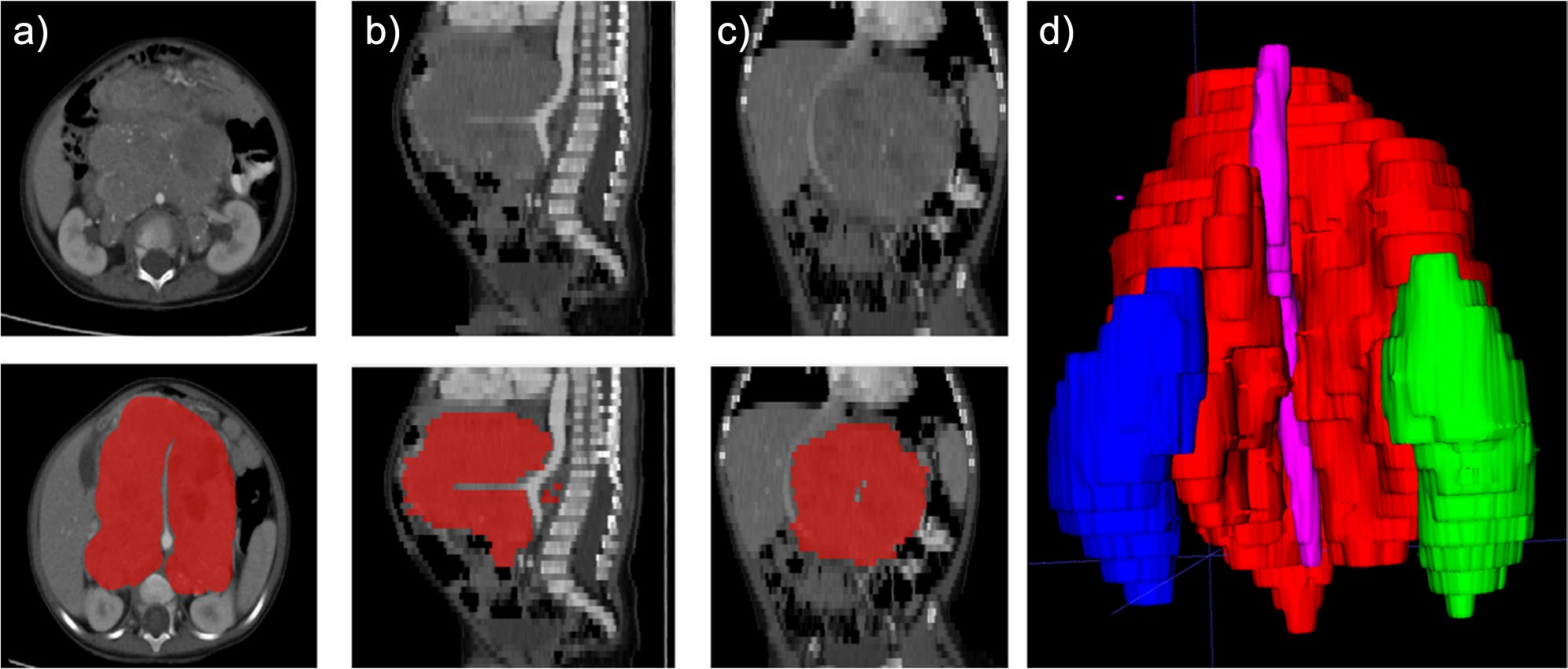
Neuroblastoma with large retroperitoneal mass. Typical characteristics such as crossing the midline, calcifications, and encasement of the vessels are evident. **a** Axial. **b** Sagittal. **c** Coronal slices with segmentation map overlays in the lower rows. **d** Posterior view of a 3D mesh of the tumor (red), left (blue), and right (green) kidneys, along with encased aorta (purple)

**Fig. 4:**
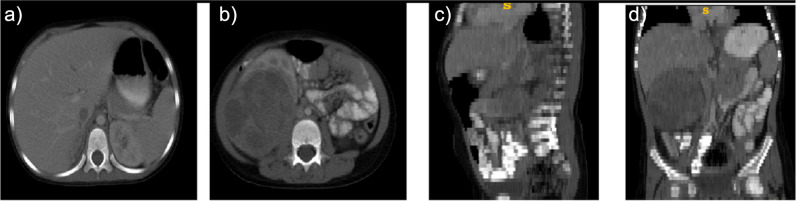
Wilms tumor with typical features. **a**, **b** Axial. **c** Sagittal. **d** Coronal slices. Tumor thrombus extending into vena cava inferior is evident in *a*. Typical heterogenous areas with large low-density necrotic zones not extending the midline in **b**, **c**, and **d**

Three radiologists (B.B., A.A.T., G.O. with 3, 5, and 10 years of experience) blinded to the clinicopathologic data performed volumetric segmentation of 60 patients using Slicer 3D [15]. This dataset is used to determine the robust feature set among different segmentations of radiologists. One radiologist (A.A.T. with 5 years of experience) completed the segmentation of the remaining patients with the same protocol (Figs. 2, 3, and 4).

The same three radiologists also examined the images after segmentation. They predicted the tumor type based on CT imaging features along with calcification, necrosis, tumor thrombus, and extension across the midline.

### Radiomic feature extraction and selection

Pyradiomics [16], an open-source Python package (v3.0 https://pyradiomics.readthedocs.io/en/latest/), was used for feature extraction. Laplacian of Gaussian filter (LoG) transformation with five distinct sigma values and one level 3D wavelet transformation (WaT) was used along with the original images. A total of 1218 features complying with Image Biomarker Standardization Initiative guidelines were extracted [16–19]. Unsupervised and supervised feature selection methods were applied sequentially. Robust, non-redundant, and high-variance features were selected for unsupervised feature selection. Four different supervised feature selection methods—statistical filter based, volcano plot based, recursive feature elimination model based, and maximum relevance minimum redundancy based—were used to leverage each method’s advantage [20–22]. More details are disclosed in the supplementary material.

For the clinical dataset, the number of features was already low; therefore, no feature selection method was applied.

### Model building and selection

**Fig. 5:**
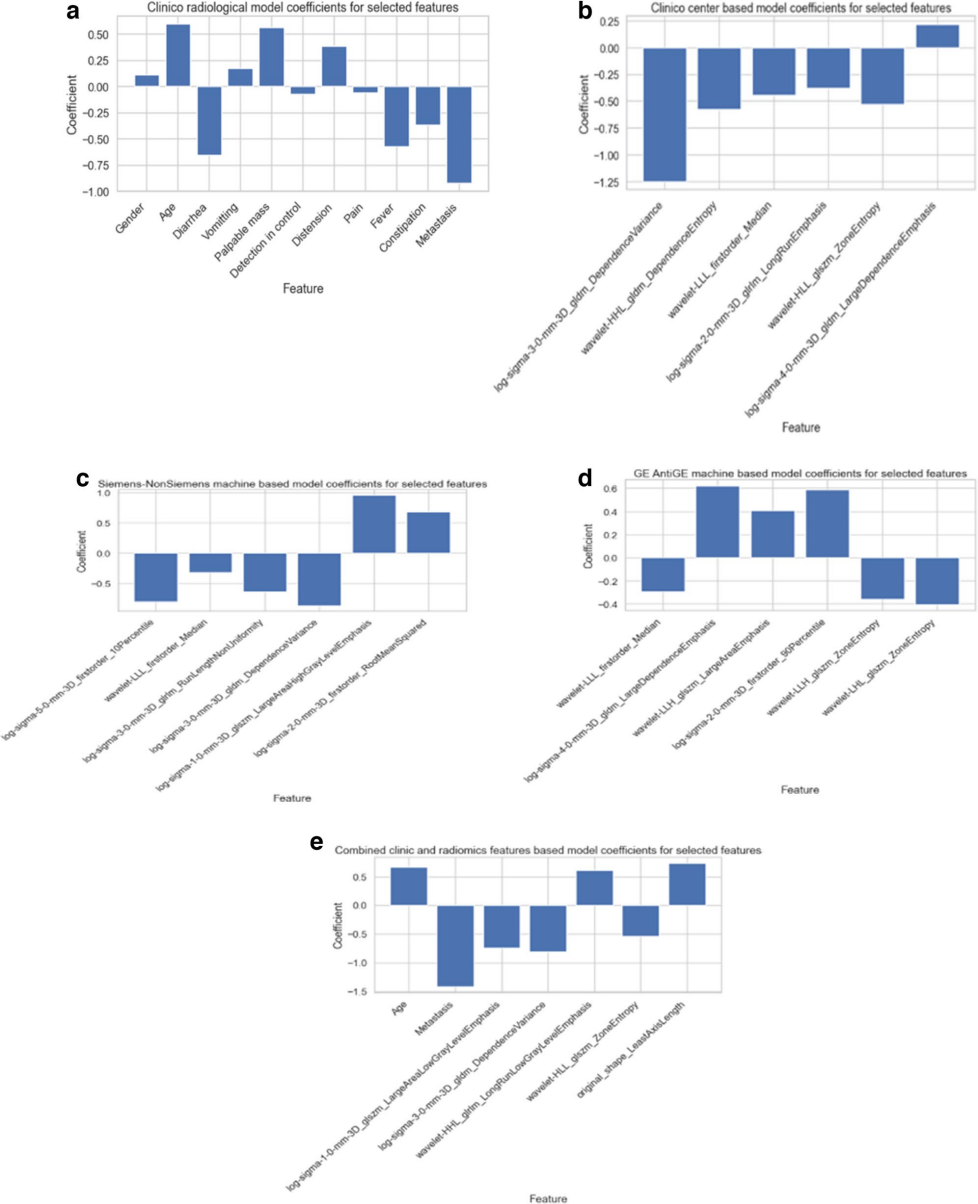
Selected features with model coefficients for each task. **a** Clinic-radiologic model. **b** Clinical center-based radiomics model. **c** Siemens vs. non-Siemens CT machine-based radiomics model. **d** GE vs. non-GE CT machine-based radiomics model. **e** Combined model

**Fig. 6:**
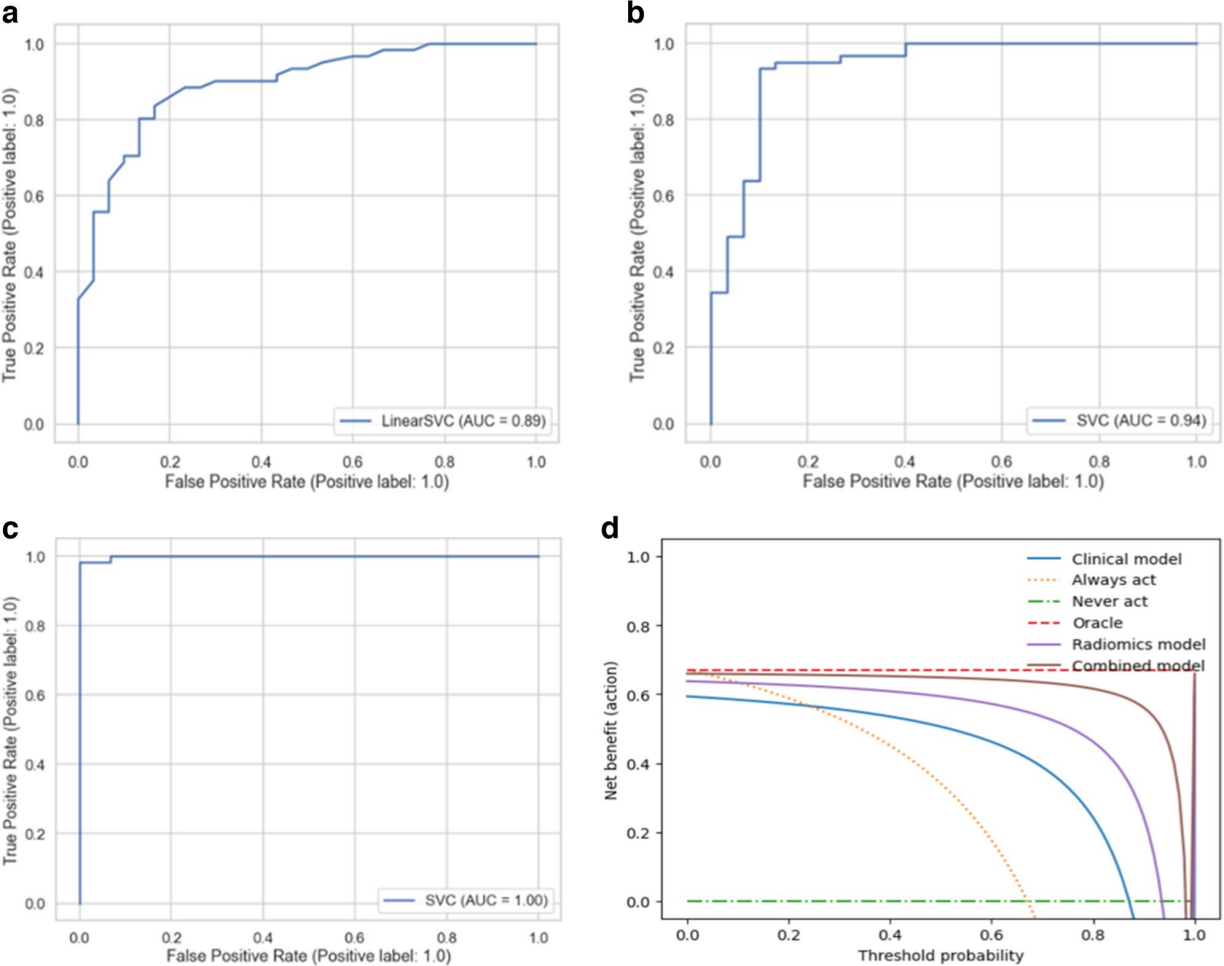
ROC curves and decision curves for the models in the training phase. **a** Clinic-radiologic model ROC curve. **b** Clinical center-based radiomics model ROC curve. **c** Combined model ROC curve. **d** Decision curve for the models in **a**, **b**, and **c**

Support vector machines (SVM) and Random Forests (RF) are successful classifier models that can be applied to both linear and non-linear classification tasks [23, 24]. SVM is a linear classifier; however, by using some kernels to project the data into higher dimensional feature space, it can be converted to a non-linear classifier. We used radial basis function (RBF) kernel for the non-linear application of SVM [23] and Synthetic Minority Oversampling Technique (SMOTE) and weighting the loss function to combat the data imbalance problem. Therefore, we experimented with seven model types (SVM, SVM with RBF, SVM with loss function weighting, SVM with SMOTE, RF, RF with loss function weighting, and RF with SMOTE) along with four feature-selection method-based feature sets. We used 10 times fourfold repeated stratified cross-validation scheme-based training to estimate the skill of the models better and reported the performance of the best models as mean and SD. An overview of the model building and training process is summarized in Fig. 1. We also provided bar plots of selected features against model coefficients (Fig. 5) and ROC curves of the models for each task (Fig. 6).

### Statistics

Python (Version 3.7.3 https://www.python.org) was used for feature extraction, selection, and statistical analysis. Matplotlib, seaborn, numPy, pandas, sciPy, and sklearn packages were employed for the analysis. The Mann–Whitney U test was used for the continuous variables (age), and the chi-square test was used for the categorical variables. Statistical significance was set at *p* < 0.05. Dice score, defined as two times intersection over the union of two segmentation masks, was used to assess the concordance of segmentation ROI among users. Cohen’s kappa was used for inter-user agreement of predictions of observers.

### Code availability

The code will be publicly available at https://github.com/ozgurkoska78/wt_nb after the manuscript is accepted.

## Results

### Patients

Of 174 patients with pathologically confirmed NB and WT patients, 57 consecutive NB and 90 consecutive WT patients were included after applying inclusion and exclusion criteria. The patients’ demographic, clinical, and radiological characteristics are summarized in Table 1. For the image acquisition center-based analysis, the training set acquired in our university hospital included 30 NB and 61 WT patients. The external validation set acquired in different centers included 27 NB and 29 WT patients.

**Table 1:** Demographic and clinical characteristics of the dataset. Counts, percentages (in the parenthesis), and statistical p values were presented

	Our center			External data		
	WT	NB	*p*	WT	NB	*p*
Gender	M: 28 (46%) F: 33 (54%)	M: 16 (53%) F: 14 (47%)	.65	M: 15 (51%) F: 14 (49%)	M: 15 (55%) F: 12 (45%)	.98
Age	< 2 y: 29 (47%) > 2 y: 32 (53%)	< 2 y: 21 (70%) > 2 y: 9 (30%)	.07	< 2 y: 11 (62%)) > 2 y: 18 (38%)	< 2 y: 14 (51%)) > 2 y: 13 (49%)	.43
Diarrhea	(−): 60 (98%) (+): 1 (2%)	(−): 27 (90%) (+): 3 (10)	.19	(−): 28 (96%) (+): 1 (4%)	(−): 26 (96%) (+): 1 (4%)	.50
Vomiting	(−): 57 (93%) (+): 4 (7%)	(−): 28 (93%) (+): 2 (7%)	.66	(−): 26 (89%) (+): 3 (11%)	(−): 24 (88%) (+): 3 (12%)	.73
Mass	(−): 38 (62%) (+): 23 (38%)	(−): 26 (86%) (+): 4 (14%)	.03	(−): 21 (72%) (+): 8 (28%)	(−): 26 (96%) (+): 1 (4%)	.03
Incidental	(−): 54 (88%) (+): 7 (12%)	(−): 26 (86%) (+): 4 (14%)	.93	(−): 26 (89%) (+): 3 (11%)	(−): 21 (77%) (+): 6 (23%)	.38
Abdominal distension	(−): 36 (59%) (+): 25 (41%)	(−): 26 (86%) (+): 4 (14%)	.01	(−): 15 (51%) (+): 14 (49%)	(−): 24 (88%) (+): 3 (12%)	.006
Abdominal Pain	(−): 54 (88%) (+): 7 (12%)	(−): 26 (86%) (+): 4 (14%)	.93	(−): 23 (79%) (+): 6 (21%)	(−): 23 (85%) (+): 4 (15%)	.82
Fever	(−): 60 (98%) (+): 1 (2%)	(−): 24 (80%) (+): 6 (20%)	.07	(−): 26 (89%) (+): 3 (11%)	(−): 25 (92%) (+): 2 (8%)	.93
Constipation	(−): 60 (98%) (+): 1 (2%)	(−): 29 (96%) (+): 1 (4%)	.8	(−): 28 (96%) (+): 1 (4%)	(−): 25 (92%) (+): 2 (8%)	.94
Metastasis	(−): 49 (80%) (+): 12 (20%)	(−): 9 (30%) (+): 21 (70%)	< .01	(−): 26 (89%) (+): 3 (11%)	(−): 14 (49%) (+): 13 (51%)	.004

*WT* Wilms tumor, *NB* neuroblastoma, *p* statistical *p* value, *M* male, *F* female, *y* age in year, ( −) feature is not present, ( +) feature is present

The NB patients tend to be younger than WT patients (NB mean age 23.77 months and WT mean age 34.78 months); however, after binarization at 24 months cut-off, there was no statistically significant difference between two groups either in center-based training or external validation sets (*p* = 0.07) There was no statistically significant difference in gender among NB and WT patients either in center-based training (NB male: 16 (53.3%), female: 14 (46.7%), WT male: 28 (45.9%), female: 33 (54.1%), *p* = 0.65) or in external validation set as well (NB male: 15 (55.5%), female: 12 (44.5%), WT male: 15 (51.7%), female: 14 (48.3%), *p* = 0.98).

Twenty different machines from four vendors were used for image acquisition. Images of 62 patients were acquired by Siemens (42%), 59 by GE (40%), 18 by Toshiba (12%), and eight by Philips (6%) CT machines.

### Interobserver correlation and human reader study

Dice scores were calculated to analyze the robustness of segmentation maps, and 0.86 mean dice scores between observers 1 and 2, 0.88 for observers 1 and 3, and 0.92 between observers 2 and 3 were obtained. The error maps of one NB patient between observers are provided in Fig. 2.

Cohen’s kappa value was calculated to analyze the observers’ agreement for tumor class prediction. The Kappa value was 0.65, interpreted as substantial agreement between raters. Observer 1 and observer 2 predicted the correct class with 0.96 accuracy. The accuracy for observer 3 was 0.93. The two patients could not be correctly predicted by either of the observers.

#### Clinical data analysis

Age, gender, and clinical symptoms and signs—including diarrhea, constipation, mass, pain, fever, failure to thrive, incidental detection, and metastasis—were used for the clinical model. With the clinical model, 0.80 mean AUC was obtained (Table 2). For the inference with the best model, 0.83 train and 0.70 external validation accuracy were obtained (Table 3).

**Table 2:** Performance metrics of the models. The metrics were derived from 40 experiments based on 10 times fourfold cross-validation for better estimation of their skills. Mean value and standard deviation in the parentheses were provided in the table

	SVM weighted		SVM RBF	RF	SVM		SVM SMOTE	RF weighted	RF SMOTE
Center based (radiomics)	F1	.90 (.05)	.89 (.04)	.89 (.04)	.89 (.04)		.87 (.06)	.88 (.06)	.86 (.04)
	Acc	.86 (.06)	.84 (.05)	.85 (.05)	.86 (.05)		.87 (.05)	.85 (.07)	.86 (.05)
	Sens	.91 (.07)	.92 (.06)	.92 (.07)	.92 (.06)		.85 (.09)	.86 (.10)	.88 (.06)
	Prec	.89 (.05)	.86 (.06)	.87 (.06)	.88 (.06)		.90 (.06)	.91 (.05)	.85 (.07)
	AUC	.91 (.05)	.85 (.05)	.90 (.06)	.91 (.04)		.93 (.05)	.90 (.05)	.93 (.06)
Machine based (Siemens) (radiomics)	F1	.80 (.07)	.81 (.07)	.77 (.06)	.84 (.05)		.83 (.07)	.74 (.07)	.76 (.08)
	Acc	.78 (.07)	.79 (.08)	.74 (.07)	.82 (.06)		.83 (.06)	.73 (.06)	.77 (.07)
	Sens	.79 (.12)	.83 (.11)	.78 (.10)	.85 (.09)		.82 (.10)	.71 (.12)	.76 (.11)
	Prec	.82 (.08)	.81 (.09)	.78 (.09)	.83 (.08)		.85 (.08)	.80 (.09)	.79 (.09)
	AUC	.89 (.07)	.86 (.06)	.81 (.07)	.90 (.06)		.92 (.06)	.82 (.07)	.85 (.06)
Machine based (GE) (radiomics)	F1	.87 (.05)	.87 (.04)	.85 (.07)	.89 (.04)		.86 (.05)	.83 (.10)	.85 (.07)
	Acc	.85 (.06)	.84 (.05)	.88 (.11)	.86 (.05)		.86 (.05)	.81 (.08)	.86 (.06)
	Sens	.88 (.09)	.91 (.06)	.88 (.11)	.91 (.08)		.87 (.08)	.81 (.14)	.84 (.11)
	Prec	.88 (.06)	.84 (.05)	.83 (.06)	.88 (.06)		.86 (.07)	.87 (.06)	.87 (.07)
	AUC	.91 (.05)	.87 (.07)	.87 (.06)	.91 (.06)		.93 (.07)	.87 (.04)	.91 (.05)
Clinical (clinic/radiologic)	F1	.81 (.06)	.80 (.07)	.80 (.06)	.78 (.04)		.81 (.06)	.76 (.08)	.77 (.05)
	Acc	.75 (.07)	.72 (.08)	.72 (.08)	.72 (.05)		.77 (.08)	.72 (.06)	.74 (.06)
	Sens	.84 (.11)	.83 (.01)	.84 (.10)	.80 (.11)		.82 (.06)	.81 (.09)	.84 (.08)
	Prec	.79 (.06)	.78 (.06)	.76 (.06)	.79 (.08)		.77 (.06)	.74 (.07)	.72 (.04)
	AUC	.80 (.07)	.79 (.09)	.74 (.09)	.76 (.06)		.79 (.08)	.81 (.12)	.76 (.08)
Combined (radiomics + clinical)	F1	.93 (.03)	.94 (.03)	.93 (.03)	.91 (.05)	.93 (.04)		.89 (.04)	.91 (.05)
	Acc	.91 (.04)	.92 (.04)	.91 (.04)	.89 (.05)	.92 (.04)		.90 (.05)	.92 (.02)
	Sens	.94 (.06)	.95 (.05)	.94 (.05)	.93 (.06)	.91 (.05)		.92 (.04)	.93 (.06)
	Prec	.93 (.04)	.94 (.04)	.92 (.05)	.93 (.05)	.94 (.03)		.90 (.04)	.91 (.06)
	AUC	.97 (.06)	.96 (.06)	.94 (.04)	.94 (.05)	.95 (.06)		.91 (.06)	.94 (.06)

*SVM* support vector machine, *RBF* radial basis function, *RF* Random Forest, *Acc* accuracy, *Sens* sensitivity, *Prec* precision, *AUC* area under the curve of receiver operator curve, *SMOTE* synthetic minority oversampling technique

**Table 3:** Performance metrics with best-performing features, model pipeline and hyperparameter combinations

	Training		External validation	
Center-based model (radiomics) MRMR selected features SVM with class weights	F1	0.92	F1	0.68
	Acc	0.91	Acc	0.67
	Sens	0.91	Sens	0.67
	Prec	0.92	Prec	0.69
	AUC	0.94	AUC	0.80
Machine-based model (Siemens) (radiomics) RFE selected features SVM	F1	0.87	F1	0.77
	Acc	0.87	Acc	0.77
	Sens	0.86	Sens	0.83
	Prec	0.87	Prec	0.79
	AUC	0.95	AUC	0.94
Machine-based model (GE) (radiomics) RFE selected features SVM	F1	0.91	F1	0.71
	Acc	0.90	Acc	0.68
	Sens	0.89	Sens	0.68
	Prec	0.91	Prec	0.70
	AUC	0.94	AUC	0.82
Combined model (clinic and radiomics) RFE selected features SVM (radial basis function)	F1	0.97	F1	0.73
	Acc	0.98	Acc	0.73
	Sens	0.97	Sens	0.72
	Prec	0.97	Prec	0.78
	AUC	0.99	AUC	0.80

#### Clinical center-based radiomics model

The model with the best F1 score was an SVM model with weighted coefficients built with MRMR-selected features. This model achieved an F1 score of 0.90 (SD:0.05) and an AUC of 0.91 (SD:0.05) (Table 2). With best model feature set combination, train accuracy was 0.92, AUC was 0.94, external validation accuracy was 0.71, and AUC was 0.80 (Table 3).

#### CT machine-based radiomics model

There were 20 NB and 42 WT patients in the Siemens-patients dataset, which served as external validation set, and 37 NB and 48 WT patients in the other machines dataset, which served as training set. Similarly, there were 23 NB and 36 WT patients in the GE-patients dataset, which served as a distinct external validation set, and 34 NB and 54 WT patients in the other machines dataset, which served as a distinct training set for the alternative classification task. With Siemens and other machines-based partitions, the most successful model was built with RFE-selected features and SVM, which achieved an F1 score of 0.84 (SD:0.05) and an AUC 0.90 (SD:0.06). Similarly, with GE and other machines-based partitions, an SVM model with RFE-selected features had the highest performance with an F1 score of 0.89 (SD:0.04) and an AUC of 0.91.

#### Clinic-radiological and radiomics combined model

We further extended our analysis by combining the clinic-radiologic and radiomics data information. For this analysis, we first created a common union radiomics dataset, which included the features selected from each of the four different feature selection strategies. This strategy yielded a 13-feature dataset. Then, we added all 10 clinic-radiologic features to obtain a combined dataset, followed by RFE-based feature reselection. With this strategy, we selected seven best features, of which two were clinic-radiologic based (age and metastasis), and five were radiomics based (two LoG-transform, two wavelet-transform, and one original shape-based features). SVM with radial basis function-based model with this feature set achieved the best overall performance with an F1 score of 0.94 (SD:0.03) and an AUC 0.96 (SD:0.04).

#### Clinical utility of the prediction models

Compared with scenarios in which no combined prediction model was used, the combined model produced a better net benefit than clinical and radiomics models for all thresholds (Fig. 6d).

### Subgroup analysis based on lesion volume

We further analyzed the effect of lesion size on the predictions of the radiomics model. We had 44 patients with lesions smaller than 150 cc and 64 patients with lesions ranging from 150–500 cc (Supplementary material Figure S3). Human observers correctly classified these small and intermediate size lesions since the organ of origin was more easily determined. In contrast, the model failed more in classifying these lesions because their smaller size introduced difficulties in finding textural patterns. By excluding these small and intermediate-size lesions, the predictive accuracy of the three human observers dropped to 0.84, 0.81, and 0.71. In contrast, the predictive accuracy of the radiomics model in the external validation set increased to 0.84. When we extended this analysis to exclusively > 1000 cc lesions, the predictive accuracy of the human observers further dropped to 0.7, 0.7, and 0.6, while that of the radiomics model on the external validation set increased to 0.9. This analysis further justified the benefit of the radiomics model as an aid to decision-making in large tumors that exhibit difficulty in finding the organ of origin.

### Radiology quality score

We further calculated radiology quality scores based on percentages. The score for our study was 50%. However, some items of radiology quality score (RQS), such as cost-effectiveness analysis, biological basis analysis, and test–retest assessment with CT, could not be applied to our study [25].

## Discussion

Although there is an increasing interest in radiomics and machine learning studies for medical imaging, there is a lack of built and validated CT radiomics models to predict neuroblastoma or Wilms tumor in solid pediatric abdominal tumors preoperatively. We constructed a machine learning-based CT clinic radiomics model to noninvasively predict neuroblastoma or Wilm’s tumor in pediatric patients in abdominal CT examinations with a mean F1 score of 0.94, 0.93 mean accuracies, and 0.96 mean AUC. We showed that incorporating clinical and radiological knowledge into the radiomics features increased the model’s performance. Importantly, two patients not correctly identified by either of the observers were correctly predicted by the developed model, which is additional evidence of the benefit of machine learning-based radiological decision support systems. Restricting the analysis to the largest tumors with a tumor volume greater than 1000 cc, which introduces the most difficulty for differential diagnosis, our models surpass the human-level performance by a significant margin of 20–30%. Radiomics has proved to be an important digital biopsy approach defining underlying histopathologic features of tumors [26, 27]. We performed hierarchical screening and reduction of radiomics features and integrated clinical and radiological knowledge to build a robust classifier. The inferior performance of the model in the external validation sets may be attributed to the heterogeneity of the dataset obtained from multiple institutions and different machines. In this cohort, 20 machines with various kernel reconstructions and acquisition parameters were included to reflect real-life conditions better. We tried to reduce the data heterogeneity by preprocessing the images, including voxel resampling into 1 mm^3^ resolution, intensity shift, and gray-level discretization. Regardless, the combined model, which integrated the imaging-based features with demographic and clinical features, demonstrated remarkable performance in predicting NB or WT. Increasing the number of samples acquired by different machines, using different protocols, and different kernel reconstructions, which we aim to carry out in future studies, might be a good strategy to mitigate the performance drop in external test sets. This approach involves modeling and learning the inherent heterogeneity of different image acquisition conditions.

Few research papers in the radiological literature deal with the differential diagnosis of renal and non-renal origin tumors using imaging methods [10, 28]. A research paper that reported the diagnostic accuracy based on these findings achieved 82% global accuracy [29]. In another study, authors stated that nine patients with NB were identified who had an exploratory laparotomy with a preoperative diagnosis of WT; eight underwent nephrectomy at exploration [28]. However, in NB, every effort should be made to preserve both kidneys [10, 28, 29]. The authors recommended checking urinary catecholamine levels, which would be present in 90% of NB if there is any question about the diagnosis [28, 29]. In our dataset, we had the urinary catecholamine records of the NB patients but not for the WT patients; thus, we did not include them in our models. Nevertheless, our model reached 93% accuracy and 96% AUC score, outperforming urinary catecholamines alone. Including the laboratory variables, such as urinary catecholamines, might further improve our result, which we plan to test in a future study.

In our combined model, seven features, of which three were age, metastasis presence, and short axis length, were selected for a final classification task. In one study similar to our findings, it was found that as volume and tumor size increase, there is a greater probability of diagnosing a WT (OR:7.93 and 4.37, respectively), and an inverse correlation between the presence of metastasis and having WT was observed (OR:0.19) [29].

Radiomics has shown promising results for several types of cancer [30–32], and more recently, it has been applied to NB [10, 11, 33–35] and WT [15]. Most of the research on radiomics for NB focuses on MYC amplification identification [10, 33–35]. These studies reported AUC scores ranging from 0.72 to 0.97 [10, 34, 35]. No external validation cohort was used in the published studies. In our study, both center-based and two different CT machine-based trained models achieved 0.82, 0.95, and 0.80 AUC scores in the external validation set, reaching 0.94 AUC in all training sets.

The 123I-MIBG is selectively concentrated in more than 90% of neuroblastomas; therefore, it can also be used to differentiate NB and WT [1]. However, the high radiation of this technique limits its utilization. The availability of this imaging facility in some centers and the additional costs related to the radiotracer should be further considered. CT is routinely acquired to evaluate the anatomical relationships of the tumor, among other benefits. Our model evaluates the readily available CT images without additional cost or intervention to the patient, providing a significant advantage over radiotracer-based methods.

Although there is no research to differentiate WT and NB from abdominal CT with machine learning, the above-discussed studies justify the successful application of radiomics approach to the diagnosis at the cellular and molecular level for WT and NB. The central dogma of radiomics states that imaging phenotypes reflect pathophysiological processes that may alter the lesions’ morphology. These complex interactions of different tissue types and pathological processes can be captured by computational tools [16]. In our study, four second-order radiomics features were selected for the final classifier; two were LoG-based, and the remaining two were WaT-based. Laplace transform takes a second-order derivative of the image, capturing sudden changes in pixel values [19]. In our study, the GLSZM Large Area Low Gray Level Emphasis (LALGLE) feature was obtained from the LoG filter with a sigma value = 1 and GLDM Dependence Variance (DV) feature from LoG with sigma value = 3 was selected for the final classification. The edges in the image represent the interfaces of different compositions. Therefore, LoG-transformed images can leverage accentuated edge information. High GLDM DV values may correspond to more similar regions; thus, homogenous texture and low values may represent local heterogeneity. GLSZM LALGLE measures the proportion in the image of the joint distribution of larger-size zones with lower gray-level values [16, 17]. We can interpret this as the model focusing on low-density zones interspersed within the gross tumor volume.

Wavelet-transform can transform the image into high and low-frequency components by applying high-pass and low-pass filters in each of the three directions, which yields eight different image sets exhibiting different combinations of high and low-frequency components in each direction [18]. Our model selected two WaT-based features: WaT HHL GLRLM Long Run Low Gray Level Emphasis (LRLGLE) and WaT HLL GLSZM Zone Entropy.

The selected feature set also indicated that our model focused on relative distributions of different tissue groups, which gives a heterogeneous appearance to the naked eye, particularly giving more importance to low-density regions.

Our study’s major limitations were dataset size and retrospective design, which may introduce selection bias. Nevertheless, 147 patients from a single center archive for a childhood cancer population is not so small, and we took great care to follow best practices in machine learning in medical image classification tasks. Another limitation was the reproducibility problem of the radiomics studies. We applied strict criteria to select robust features under different user segmentations. However, the value of selected features should be validated in more extensive and heterogeneous cohorts. Last, we could not integrate some important clinical and radiological variables such as urinary catecholamine levels, presence of tumor thrombus, encasement of vessels, and presence of calcification since the information was collected for one class, not both. In a future study, we plan to integrate richer clinical and radiological variables and more advanced feature engineering for better performance.

## Conclusion

In conclusion, the CT-based clinic radiologic radiomics combined model could noninvasively predict Wilms tumor or neuroblastoma preoperatively. Notably, that model correctly predicted two patients, which none of the radiologists could correctly predict. This model may serve as a noninvasive preoperative predictor of neuroblastoma/Wilms tumor differentiation in CT as a decision support tool, which should be further validated in large prospective models.

## Supplementary Information

Below is the link to the electronic supplementary material.

Supplementary file1 (PDF 564 KB)

## Abbreviations

GLDM: Gray level dependency matrix
GLSZM: Gray level size zone matrix
LoG: Laplace of gaussian
NB: Neuroblastoma
RBF: Radial basis function
RF: Random Forest
RFE: Recursive feature elimination
SMOTE: Synthetic minority oversampling technique
SVM: Support vector machine
WaT: Wavelet transform
WT: Wilms tumor

## Declarations

### Guarantor

The scientific guarantor of this publication is Dr. İlker Özgür Koska.

### Statistics and biometry

Dr. İlker Özgür Koska has significant statistical expertise.

### Study subjects or cohorts overlap

None of the study subjects or cohorts have been previously reported in any journal or publication.

### Methodology

• Retrospective

• Case–control study

• Performed at one institution

## References

[1] Maris JM, Hogarty MD, Bagatell R, Cohn SL (2007) Neuroblastoma. Lancet. 10.1016/S0140-6736(07)60983-0 17586306 10.1016/S0140-6736(07)60983-0

[2] Davidoff AM (2012) Wilms tumor. Adv Pediatr. 10.1016/j.yapd.2012.04.001 22789581 10.1016/j.yapd.2012.04.001 PMC3589819

[3] Dumba M, Jawad N, McHugh K (2015) Neuroblastoma and nephroblastoma: a radiological review. Cancer Imaging. 10.1186/s40644-015-0040-6 25889326 10.1186/s40644-015-0040-6 PMC4446071

[4] McHugh K (2007) Renal and adrenal tumours in children. Cancer Imaging. 10.1102/1470-7330.2007.0007 17339140 10.1102/1470-7330.2007.0007 PMC1828369

[5] Vujanić GM, Gessler M, Ooms AHAG et al (2018) The UMBRELLA SIOP-RTSG 2016 Wilms tumour pathology and molecular biology protocol. Nat Rev Urol. 10.1038/s41585-018-0100-3 30310143 10.1038/s41585-018-0100-3 PMC7136175

[6] de Carvalho LG, Kobayashi T, Cypriano MDS et al (2021) Diagnostic errors in Wilms’ tumors: learning from our mistakes. Front Pediatr. 10.3389/fped.2021.757377 34760854 10.3389/fped.2021.757377 PMC8573411

[7] Kaste SC, Dome JS, Babyn PS et al (2008) Wilms tumour: prognostic factors, staging, therapy and late effects. Pediatr Radiol. 10.1007/s00247-007-0687-7 18026723 10.1007/s00247-007-0687-7

[8] Jackson TJ, Williams RD, Brok J et al (2019) The diagnostic accuracy and clinical utility of pediatric renal tumor biopsy: report of the UK experience in the SIOP UK WT 2001 trial. Pediatr Blood Cancer. 10.1002/pbc.27627 30761727 10.1002/pbc.27627 PMC6522371

[9] Strenger V, Kerbl R, Dornbusch HJ et al (2007) Diagnostic and prognostic impact of urinary catecholamines in neuroblastoma patients. Pediatr Blood Cancer. 10.1002/pbc.20888 16732582 10.1002/pbc.20888

[10] Wu H, Wu C, Zheng H et al (2021) Radiogenomics of neuroblastoma in pediatric patients: CT-based radiomics signature in predicting MYCN amplification. Eur Radiol. 10.1007/s00330-020-07246-1 33118047 10.1007/s00330-020-07246-1

[11] Brisse HJ, Blanc T, Schleiermacher G et al (2017) Radiogenomics of neuroblastomas: relationships between imaging phenotypes, tumor genomic profile and survival. PLoS One. 10.1371/journal.pone.0185190 28945781 10.1371/journal.pone.0185190 PMC5612658

[12] Tan E, Merchant K, Kn BP et al (2022) CT-based morphologic and radiomics features for the classification of MYCN gene amplification status in pediatric neuroblastoma. Childs Nerv Syst. 10.1007/s00381-022-05534-3 35460355 10.1007/s00381-022-05534-3

[13] Wang H, Xie M, Chen X et al (2023) Radiomics analysis of contrast-enhanced computed tomography in predicting the International Neuroblastoma Pathology Classification in neuroblastoma. Insights Imaging. 10.1186/s13244-023-01418-5 37316589 10.1186/s13244-023-01418-5 PMC10267098

[14] Ma XH, Shu L, Jia X et al (2022) Machine learning-based CT radiomics method for identifying the stage of Wilms tumor in children. Front Pediatr. 10.3389/fped.2022.873035 35676904 10.3389/fped.2022.873035 PMC9168275

[15] Kikinis R, Pieper SD, Vosburgh KG (2014) 3D Slicer: a platform for subject-specific image analysis, visualization, and clinical support. In: Jolesz, F. (eds) Intraoperative imaging and image-guided therapy. Springer, New York, NY. 10.1007/978-1-4614-7657-3_19

[16] Van Griethuysen JJM, Fedorov A, Parmar C et al (2017) Computational radiomics system to decode the radiographic phenotype. Cancer Res. 10.1158/0008-5472.CAN-17-0339 29092951 10.1158/0008-5472.CAN-17-0339 PMC5672828

[17] Zwanenburg A, Vallières M, Abdalah MA et al (2020) The image biomarker standardization initiative: standardized quantitative radiomics for high-throughput image-based phenotyping. Radiology. 10.1148/radiol.2020191145 32154773 10.1148/radiol.2020191145 PMC7193906

[18] Addison PS (2016) The illustrated wavelet transform handbook: introductory theory and applications in science, engineering, medicine and finance, Second Edition (2nd ed.). CRC Press, 99–116.

[19] Poularikas AD (2010) Transforms and applications handbook (3rd ed.). CRC Press, 199–212

[20] Chandrashekar G, Sahin F (2014) A survey on feature selection methods. Comput Electr Eng. 10.1016/j.compeleceng.2013.11.024

[21] Kumar V, Minz S (2014) Feature selection: a literature review. Smart Comput Rev. 10.6029/smartcr.2014.03.007

[22] El Aboudi N, Benhlima L (2016) Review on wrapper feature selection approaches. 2016 International Conference on Engineering & MIS (ICEMIS). 10.1109/ICEMIS.2016.7745366

[23] Cortes C, Vapnik V (1995) Support-vector networks. Mach Learn. 10.1007/BF00994018

[24] Ho TK (1995) Random decision forest. Proceedings of the 3rd International Conference on Document Analysis and Recognition, Montreal, 14–16 August 1995. 10.1109/ICDAR.1995.598994

[25] Lambin P, Leijenaar R, Deist T et al (2017) Radiomics: the bridge between medical imaging and personalized medicine. Nat Rev Clin Oncol. 10.1038/nrclinonc.2017.141 28975929 10.1038/nrclinonc.2017.141

[26] Feng Z, Li H, Liu Q et al (2023) CT radiomics to predict macrotrabecular-massive subtype and immune status in hepatocellular carcinoma. Radiology. 10.1148/radiol.221291 36511807 10.1148/radiol.221291

[27] Limkin EJ, Sun R, Dercle L et al (2017) Promises and challenges for the implementation of computational medical imaging (radiomics) in oncology. Ann Oncol. 10.1093/annonc/mdx034 28168275 10.1093/annonc/mdx034

[28] Dickson PV, Sims TL, Streck CJ et al (2008) Avoiding misdiagnosing neuroblastoma as Wilms tumor. J Pediatr Surg. 10.1016/j.jpedsurg.2008.02.047 18558200 10.1016/j.jpedsurg.2008.02.047 PMC3214966

[29] Royero-Arias MR, Salazar-Díaz LC, Moreno-Gómez LA (2021) Wilms or non-Wilms tumors? Imaging features of renal tumors in pediatrics. Rev Fac Med. 10.15446/revfacmed.v70n1.88323

[30] Silva F, Pereira T, Morgado J et al (2021) EGFR assessment in lung cancer CT images: analysis of local and holistic regions of interest using deep unsupervised transfer learning. IEEE Access. 10.1109/ACCESS.2021.307070134532201

[31] Grimm LJ, Mazurowski MA (2020) Breast cancer radiogenomics: current status and future directions. Acad Radiol. 10.1016/j.acra.2019.09.012 31818385 10.1016/j.acra.2019.09.012

[32] Smith CP, Czarniecki M, Mehralivand S et al (2019) Radiomics and radiogenomics of prostate cancer. Abdom Radiol (NY). 10.1007/s00261-018-1660-7 29926137 10.1007/s00261-018-1660-7

[33] Pereira T, Silva F, Claro P et al (2022) A random forest-based classifier for MYCN status prediction in neuroblastoma using CT images. Annu Int Conf IEEE Eng Med Biol Soc. 10.1109/EMBC48229.2022.9871349 36086471 10.1109/EMBC48229.2022.9871349

[34] Di Giannatale A, Di Paolo PL, Curione D et al (2021) Radiogenomics prediction for MYCN amplification in neuroblastoma: a hypothesis generating study. Pediatr Blood Cancer. 10.1002/pbc.29110 34003574 10.1002/pbc.29110

[35] Chen X, Wang H, Huang K et al (2021) CT-based radiomics signature with machine learning predicts MYCN amplification in pediatric abdominal neuroblastoma. Front Oncol. 10.3389/fonc.2021.687884 34109133 10.3389/fonc.2021.687884 PMC8181422

