# Supplementary material for: Radiomics in differential diagnosis of Wilms tumor and neuroblastoma with adrenal location in children
Source: Eur Radiol. 2024 Feb 5;34(8):5016–27. doi: 10.1007/s00330-024-10589-8 (PMC11255001; doi:10.1007/s00330-024-10589-8)
Supplement: Supplementary file 1 — Supplementary file1 (PDF 564 KB) [file 330_2024_10589_MOESM1_ESM.pdf]

Radiomics in Differential Diagnosis of Wilms Tumor and Neuroblastoma with Adrenal Location in Children

Electronic Supplementary Material (ESM)

Eur Radiol (2024) Koska IO, Ozcan HN, Tan AA et al.

Supplementary Material:

List of CT machines used in the study:

Siemens, Somatom Definition, Erlangen, Germany.

Siemens, Somatom, Perspective, Erlangen, Germany.

Siemens, Somatom, Scope, Erlangen, Germany.

Siemens, Somatom, Force, Erlangen, Germany.

Siemens, Sensation 16, Erlangen, Germany.

Siemens, Sensation 64, Erlangen, Germany.

Siemens, Emotion 16, Erlangen, Germany.

Siemens, Sprit, Erlangen, Germany.

GE Medical Systems, Light Speed 16, Milwaukee, WI, USA.

GE Medical Systems, Revolution Evo, Milwaukee, WI, USA.

GE Medical Systems, Revolution GSI, Milwaukee, WI, USA.

GE Medical Systems, Optima CT660, Milwaukee, WI, USA.

GE medical Systems, Brivo CT385 Series, Milwaukee, WI, USA.

Canon Medical Systems, Alexion, Otawara, Japan.

Canon Medical Systems, Activion 16, Otawara, Japan.

Canon Medical Systems, Aquilon Prime, Otawara, Japan.

Canon Medical Systems, Aquilon ONE, Otawara, Japan.

Canon Medical Systems, Aqullion Lightning, Otawara, Japan.

Philips Brillance 64, Eindhoven, Netherlands.

Philips Brillance 16, Eindhoven, Netherlands.

Details of CT machine settings and lesion volumes:

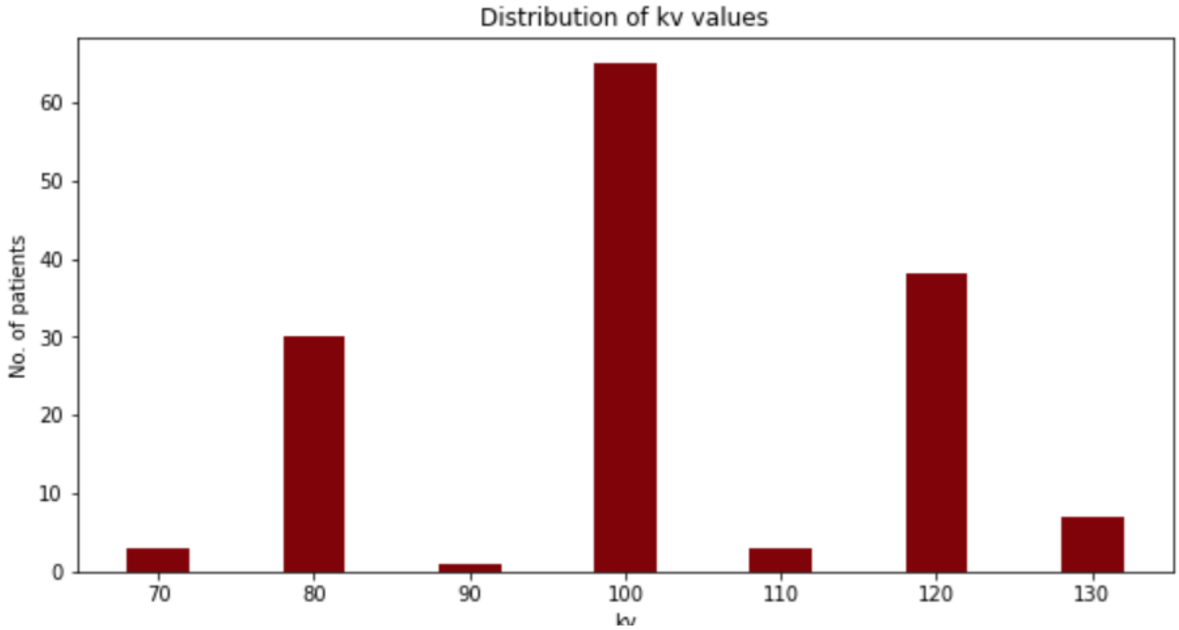

Figure S1: Bar plot of kV distribution of CT machines

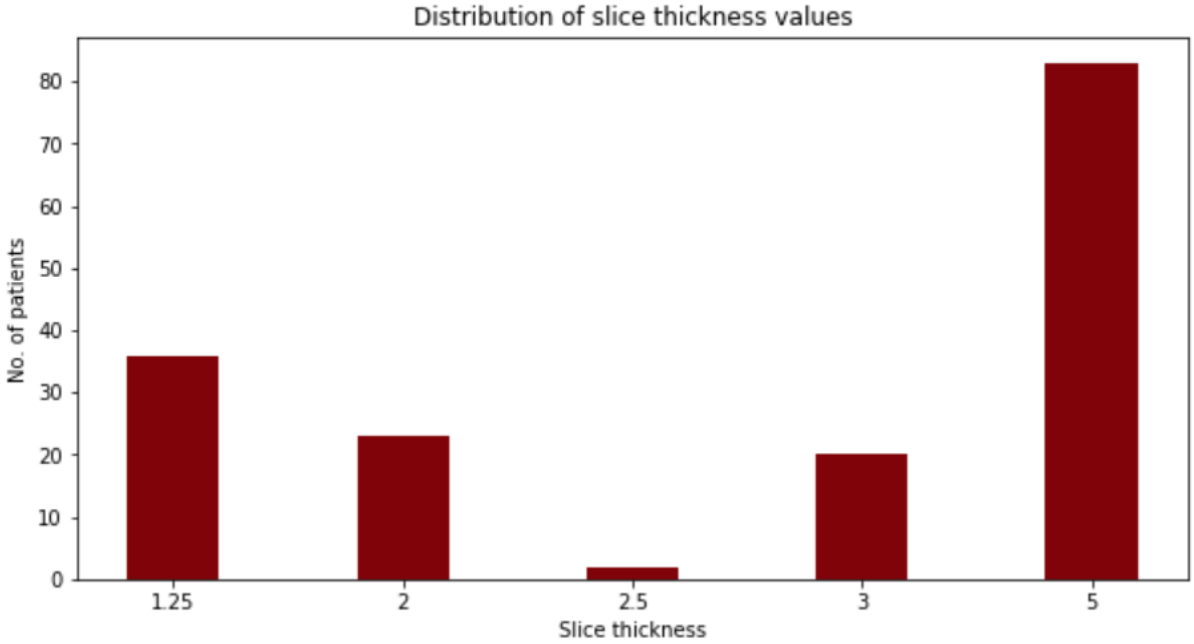

Figure S2: Bar plot of slice thickness distribution which were used in the image acquisitions.

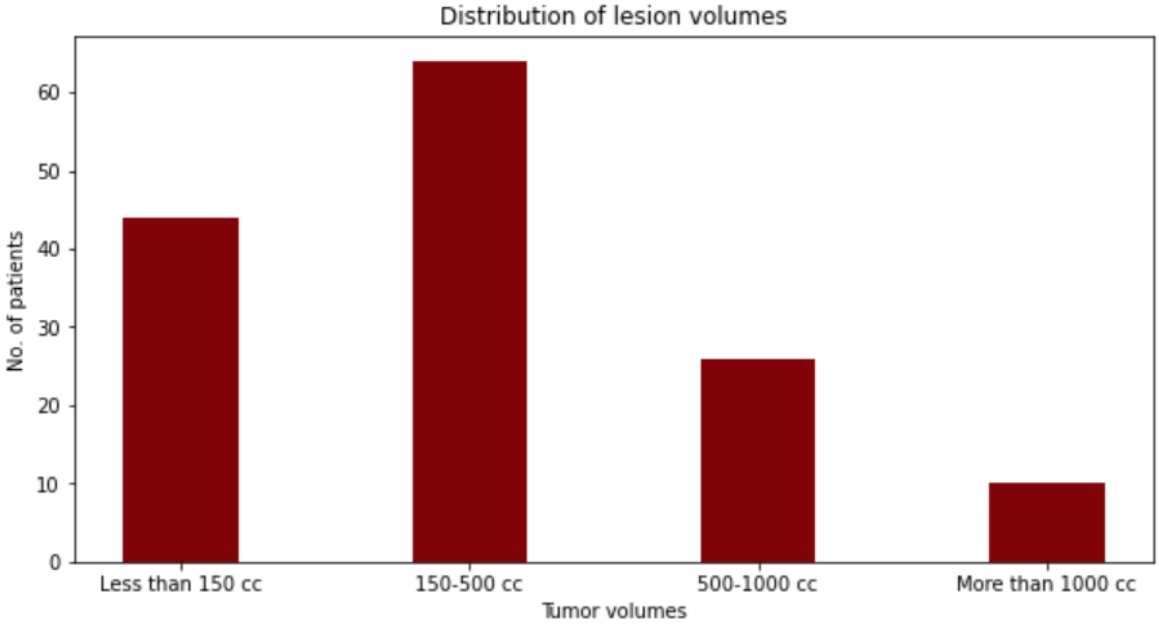

Figure S3: Bar plot of distribution of lesion volumes

Details of ROI selection, image preprocessing and feature selection:

First, density thresholding was applied to limit the affected pixel range, and then manual segmentation was applied using an appropriate size brush slice wise in Slicer 3D.

Voxels were resampled into 1x1x1 mm resolution by a cubic b-spline algorithm to correct acquisition related variations and discretized into a bin width of 25 HU followed by normalization with a normalized scale of 300.

We extracted 1218 radiomics features using the pyradiomics package [17]. Laplacian of Gaussian filter (LoG) transformation with 5 distinct sigma values and one level 3D wavelet transformation (WaT) was used along with original images (Figure S4) [19, 20]. First order statistics, shape-based 3D, Gray Level Co-occurrence Matrix (GLCM), Gray Level Run Length Matrix (GLRLRM), Gray Level Size Zone Matrix (GLSZM), and Gray Level Dependence Matrix (GLDM) based features were extracted complying with IBSI guidelines yielding 1218 features

[17, 18]. IBSI and pyradiomics documentation are good resources for a detailed explanation of these features and feature extraction methods [17, 18].

First, robust features were selected using an intersection set of cross correlation of the feature sets based on segmentations of three radiologists. The correlation cut-off was set to 0.9, and the features correlating this threshold among three datasets remained while others were discarded. This is important to ensure that the selected features can be reproduced under different user segmentation ROIs. Next, the robust features were tested for redundancy. Multicollinearity is a problem with high dimensional data sets, leading to spurious behaviors of selected features [21]. Therefore, we need to discard redundant features. Cross correlation of the features with themselves was used for this selection. The features with correlation coefficients below 0.9 were kept, and those above 0.9 were discarded to obtain a robust and nonredundant feature set. There is no consensus for selecting the cut-off value for correlation either for robustness or multicollinearity analysis. However, 0.75-0.9 is the usual range, further determined by the desired strictness of the research team [21, 22, 23]. We advocated higher reproducibility of the features under segmentation differences and selected a relatively high threshold to eliminate non-robust features. On the other hand, we preferred a more relaxed cut-off to eliminate redundant features since some redundancy could be tolerated to provide a richer feature set for further supervised feature selection steps.

The next step for unsupervised feature selection was the application of variance thresholding. The features with low variance among patients had little impact on the classification task [21, 22, 23].

Therefore, eliminating them can also help decrease the dimension of the dataset. The threshold was set as .05, eliminating features with a variance lower than 5% among patients. After these steps, supervised feature selection was applied to the pruned dataset.

After robustness analysis, the number of features consistent among different segmentations of three observers dropped to 633. After multicollinearity and low variance elimination, the following unsupervised feature selection steps yielded 134 and 64 features. With this dataset, four different supervised feature selection algorithms, which were maximum relevance minimum redundancy (MRMR), recursive feature elimination (RFE), statistical filter, and Volcano plot based, and 7 different model architectures based on SVM and RF along with different unbalanced dataset correction strategies were employed.

Using unsupervised feature selection methods for more reproducible and robust analysis, including eliminating unstable, redundant, and low variance features, the Siemens based dataset dropped to 68, and the GE based dataset dropped to 62 features. Finally, a robust, non-redundant, high variance and relevant feature set was obtained for model training.

Details of model training:

Our dataset was imbalanced with an abundance of WT patients. For an imbalanced setting, there was a set of options that could correct the tendency of the model to predict the majority class. Under-sampling the majority class, oversampling the minority class, weighting the loss function to focus on the minority class, and synthetic data generation are among them. We used weighting the loss function and SMOTE for the data imbalance problem.

SMOTE is a technique to generate data points in feature space for the minority class, which is based on computing a new point on the Euclidian distance of two preexisting points.

Cross validation (CV) is a useful technique in data-limited conditions to estimate the model performance better. The data is partitioned into k parts, and training is applied to k-1 parts and tested on the reserved one part. This process is repeated k times, in which k denotes the number of partitions the research team decided based on the number of samples and the

task. A critical point for this data partition is respecting the class distribution of the samples. To preserve class distribution in each data fold, the python scikit-learn package has a Stratified K-fold cross validation function, which we decided to use for our experiments. We applied 10 times 4-fold cross validation for our analysis.

Data leakage is an important problem for machine learning projects. There are several important steps to avoid data leakage. The train test split should be separate in every step of the experiments, including standardization of features, supervised feature selection, and actual training to avoid leaks. We used the pipeline function of the scikit-learn package and employed feature selection and standardization just after the data partition for CV. We also applied feature selection and standardization to the test partition before training to ensure there was no data leakage.

Details of selected radiomics features discussion:

Laplace transform takes the image's second order derivative, keeping strong change and discards monotonous knowledge. The strong change in pixel values corresponds to edges. However, the Laplace transform is very sensitive to noise; therefore, it should be reduced before its application. For this purpose, the image is passed from a Gaussian filter, which smooths the image and suppresses the noise. The sigma of the Gaussian filter determines the noise suppression amount. Higher sigma values will smooth the image, and only more substantial edges will be captured by the LoG filter [20]. We used 5 different sigma levels starting from 1 to 5 for the LoG transformation of the images.

A gray level dependency is defined as the number of connected voxels within a predefined distance that is the same on each center voxel [17, 18]. GLDM quantifies gray level dependencies in an image [17]. Dependency variance (DV) measures the variance in

dependence size in the image. GLSZM quantifies gray level zones in an image. A gray level zone is defined as the number of connected voxels that share the same gray level intensity. It counts the same voxel values connected (directly adjacent) irrespective of a predefined central voxel. GLSZM zone entropy (ZE) measures the uncertainty/randomness in the distribution of zone sizes and gray levels. A higher value indicates more heterogeneity in the texture patterns. GLRLM quantifies gray level runs, defined as the length in the number of pixels of consecutive pixels with the same gray level value. LRLGLRE measures the joint distribution of long run lengths with lower gray-level values [17, 18].

The selected features can be displayed as voxel-wise feature maps, which represent the lesions differently. In voxel-wise maps a small window is traversed over the image, capturing local radiomics characteristics that can be used to examine and discriminate the lesions visually (Figure S5).

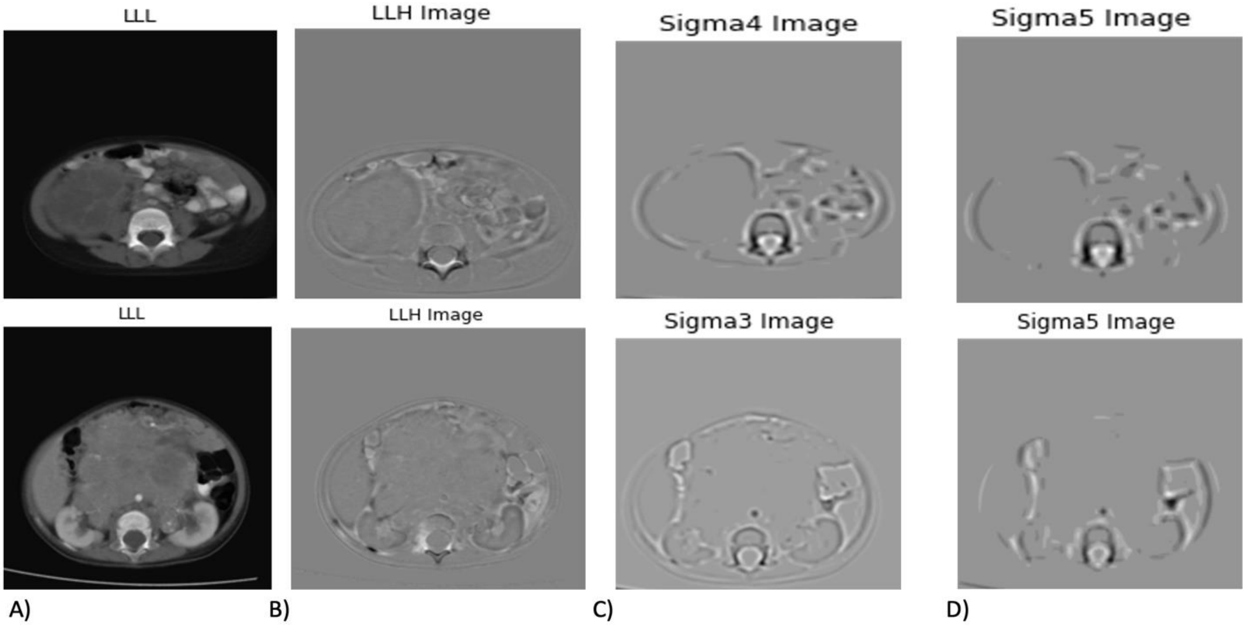

Figure S4: Upper row WT, lower row NB patient. A: Wavelet transform images with low frequency components in 3 directions. B: Wavelet transform images with low frequency

components in anteroposterior and mediolateral direction and high frequency components in supero-inferior direction. C and D LoG transformation of images with different sigma levels, which focus on edges of different strengths.

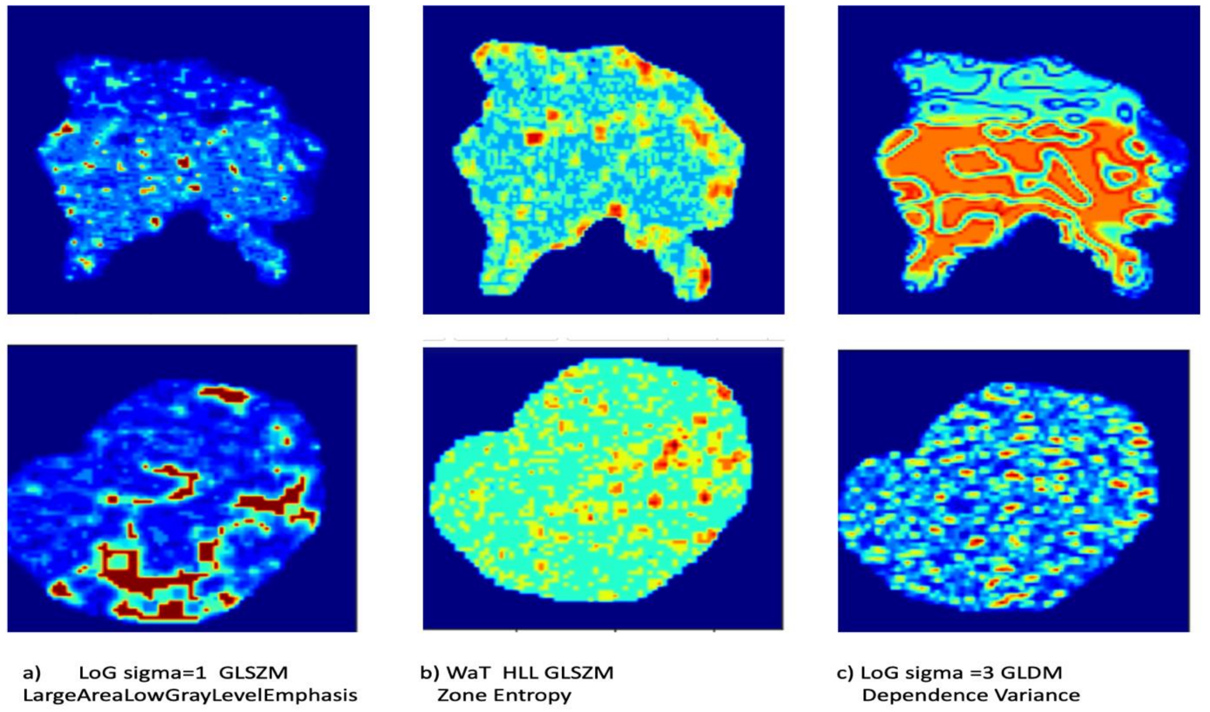

Figure S5: Voxel-wise feature maps of selected features for NB (upper row) and WT (lower row) patients.

Image protocol quality - well-documented image protocols (for example, contrast, slice thickness, energy, etc.) and/or usage of public image protocols allow reproducibility/replicability

- [x] protocols well documented
- [ ] public protocol used
- [ ] none

Multiple segmentations - possible actions are: segmentation by different physicians/algorithms/software, perturbing segmentations by (random) noise, segmentation at different breathing cycles. Analyse feature robustness to segmentation variabilities

- (●) yes
- ( ) no

Phantom study on all scanners - detect inter-scanner differences and vendor-dependent features. Analyse feature robustness to these sources of variability

- (●) yes
- ( ) no

Imaging at multiple time points - collect images of individuals at additional time points. Analyse feature robustness to temporal variabilities (for example, organ movement, organ expansion/shrinkage)

- ( ) yes
- (●) no

Feature reduction or adjustment for multiple testing - decreases the risk of overfitting. Overfitting is inevitable if the number of features exceeds the number of samples. Consider feature robustness when selecting features

- (●) Either measure is implemented
- ( ) Neither measure is implemented

Multivariable analysis with non radiomics features (for example, EGFR mutation) - is expected to provide a more holistic model. Permits correlating/inferencing between radiomics and non radiomics features

- (●) yes
- ( ) no

Detect and discuss biological correlates - demonstration of phenotypic differences (possibly associated with underlying gene–protein expression patterns) deepens understanding of radiomics and biology

- ( ) yes
- (●) no

Cut-off analyses - determine risk groups by either the median, a previously published cut-off or report a continuous risk variable. Reduces the risk of reporting overly optimistic results

- (●) yes

○ no

Discrimination statistics - report discrimination statistics (for example, C-statistic, ROC curve, AUC) and their statistical significance (for example, p-values, confidence intervals). One can also apply resampling method (for example, bootstrapping, cross-validation)

- [x] a discrimination statistic and its statistical significance are reported
- [x] a resampling method technique is also applied
- [ ] none

Calibration statistics - report calibration statistics (for example, Calibration-in-the-large/slope, calibration plots) and their statistical significance (for example, P-values, confidence intervals). One can also apply resampling method (for example, bootstrapping, cross-validation)

- [ ] a calibration statistic and its statistical significance are reported
- [x] a resampling method technique is applied
- [ ] none

Prospective study registered in a trial database - provides the highest level of evidence supporting the clinical validity and usefulness of the radiomics biomarker

○ yes

◉ no

Validation - the validation is performed without retraining and without adaptation of the cut-off value, provides crucial information with regard to credible clinical performance

- [ ] No validation
- [x] validation is based on a dataset from the same institute
- [ ] validation is based on a dataset from another institute
- [ ] validation is based on two datasets from two distinct institutes
- [ ] the study validates a previously published signature
- [ ] validation is based on three or more datasets from distinct institutes

Comparison to 'gold standard' - assess the extent to which the model agrees with/is superior to the current 'gold standard' method (for example, TNM-staging for survival prediction). This comparison shows the added value of radiomics

◉ yes

○ no

Potential clinical utility - report on the current and potential application of the model in a clinical setting (for example, decision curve analysis).

◉ yes

○ no

Cost-effectiveness analysis - report on the cost-effectiveness of the clinical application (for example, QALYs generated)

○ yes

◉ no

Open science and data - make code and data publicly available. Open science facilitates knowledge transfer and reproducibility of the study

- [ ] scans are open source
- [ ] region of interest segmentations are open source
- [x] the code is open sourced
- [ ] radiomics features are calculated on a set of representative ROIs and the calculated features and representative ROIs are open source

Total score **18** (50.00%)
